# Commentary: Comparison of historical medical spending patterns among the BRICS and G7

**DOI:** 10.3389/fphar.2016.00213

**Published:** 2016-07-14

**Authors:** Sandra C. Buttigieg, Simon Grima, Carl Camilleri

**Affiliations:** ^1^Department of Health Services Management, Faculty of Health Sciences, University of MaltaMsida, Malta; ^2^School of Social Policy, College of Social Sciences, University of BirminghamBirmingham, UK; ^3^Aston Business School, Aston UniversityBirmingham, UK; ^4^Department of Insurance, Faculty of Economics, Management and Accountancy, University of MaltaMsida, Malta; ^5^Department of Economics, Faculty of Economics, Management and Accountancy, University of MaltaMsida, Malta

**Keywords:** BRICS, G7, medical spending, justice, ethics, medical

Jakovljevic's comparison of historical medical spending patterns between BRICS and G7 groups of countries provides a highly valid contribution both from a group, as well as from an individual nation perspective (Jakovljevic, 2015). The author considers two very diverse groups of countries in terms of their wealth, population demographic profiles, as well as population health, health system needs and medical spending patterns.

The first perspective is of an ethical nature and argued through the lens of the principles of distributive justice intended for prioritizing populations on the basis of context, and in relation to allocation of scarce healthcare resources. Two principles, often used interchangeably, are the egalitarian and the equity principles. The former dictates serving those in need equally, whereas the latter consents for contextual differences so that resources are allocated fairly and justly (Persad et al., 2009; Hankins et al., 2015). In particular, equity justifies access to healthcare in that utilization should echo the populations' real needs and aims to eliminate socio-economic and other obstacles. The comparison of the two groups provides a picture of global wealth inequality that is likewise reflected in medical spending. It is therefore worth looking at additional demographic and economic indicators as shown in Table 1 to gain a more holistic understanding of the inequalities.

**Table 1:** **Demographic and economic indicators in BRICS and G7 groups of countries**.

**Demographic and economic indicators**	**Groups of Countries**	
	**BRICS**	**G7**
Description of economy	Five major emerging yet large and fast-growing national economies (Sui and Sun, 2016)	Seven major advanced economies as acknowledged by IMF (World Economic Outlook, 2016)
Population size	3 billion people or 42% of the world population (World Economic Outlook, 2016)	750.47 million people or 10% of the world population (Group of 7 countries (G7): Statistical Profile, 2016)
Combined nominal GDP	Approximately 20% of the gross world GDP (World Economic Outlook, 2016)	Approximately 39% of the gross world GDP (Group of 7 countries (G7): Statistical Profile, 2016)
Average total per capita health spending	$1004 Purchase Power Parity (PPP) in 2013 (Jakovljevic, 2015)	$4747 PPP in 2013 (Jakovljevic, 2015)

The information in Table 1 clearly shows that the egalitarian principle is not supported in view of the gross global wealth inequality in relation to the populations served; a situation that spills over to healthcare. More challenging is upholding the equity principle in that one assumes that the BRICS populations' real needs far outweigh those of G7 countries, yet their share of Total Health Expenditure (THE) as % of GDP is far less. Furthermore, the BRICS upward trend is mainly dominated by China's foremost contribution to medical spending trends worldwide. The increasing ratio of THE as a % of GDP is an indication that the BRICS countries' health expenditure is growing at a fast rate. Furthermore, one might question the meaning of real need. Population aging and chronic non-communicable diseases, referred to as “prosperity” diseases, appear to be key expensive drivers for G7 countries. However, Jakovljevic also records a historical delay of these drivers on THE in BRICS countries, which may justify the higher spending in G7 countries.

From a public health perspective, it is also wise to apply the widely used utilitarian principle of distributive justice when allocating scarce medical resources, namely to achieve maximum benefit to the maximum percentage of the world population. Conducting cost-effectiveness analysis is important to choose the most effective health policy, albeit potentially conflicting with ethical principles that do not rely on the monetary value. This principle is however difficult to assess in the report without delving deeper into the countries' disease burden and characteristics. Moreover, the report challenges the readership to question the attainability of WHO's “Health for all-all for health!” (Hong, 2014) by emphasizing the importance of long-term macro-economic analysis and its influence on medical spending. Indeed, the appreciation of the economic viewpoint enables international health organizations and health policy makers to jointly reach more meaningful explanations of the differences worldwide when formulating policies on the provision and utilization of healthcare resources and on overcoming health inequalities worldwide.

The second perspective focuses on the downward trend in all G7 countries' share of THE to global THE in 1995-2013, in contrast to the upward trend of the BRICS (with the exception of South Africa). This raises hope that the gap in THE between the two groups is diminishing. However, the stark differences in % points for individual countries' THE to global THE spells out the reality that the gap is expected to remain wide for the foreseeable future. Nevertheless, this report provides a rather enlightening picture that “successful health reforms in leading markets such as BRICS reveal a reshaping of their medical care-related expenditures” (p. 70). Despite the “bold increase in national health spending across the globe” (p. 70), the report highlights medical innovation, rising public expectations and extended longevity to explain the accelerated growth in health care spending over time. Additionally, the author discusses the two groups' population demographic differences (aging vs. younger nations) within the context of access to medical care and population needs for healthcare. However, as the author's discussion indicates, these statistics alone do not reveal sufficient information about whether spending is allocated appropriately and efficiently. Therefore, further studies are needed to determine and quantify the effect of these behavioral drivers in particular to help curb wastage and misuse/abuse of scarce resources. Additionally, barriers to global dissemination, adoption and implementation of medical innovation, from G7 (often considered as world leaders in this sector) (Kesselheim et al., 2013; Ma et al., 2014) to BRICS countries must be researched. Furthermore, even after accounting for demographic differences, public expectations in different nations/regions, e.g., North America rather than in groups of countries may help to explain higher levels of medical spending. This calls for a better understanding and quantifying of the value of needs vs. demands and their drivers.

Finally, the report uses single source data to facilitate comparisons. Additionally, the author considers 19 years of observations as insufficient to uncover long-term THE trends. A number of factors, which are usually expected to affect THE of a country tend to vary over a long period rather than a short period of time (e.g., changing population structures). The effect of such factors might not be fully captured if the data set being used is of a relatively short time frame. However, the counter-argument is that longer periods may distort findings because of larger variations over time particularly in medical and information technology innovations that distinguished this period from the previous decades.

## Author contributions

All three authors contributed actively in the development and finalization of this general commentary. The corresponding author provided the first draft after a group discussion with the two co-authors providing comments and edits until we reached consensus on the final version for submission.

### Conflict of interest statement

The authors declare that the research was conducted in the absence of any commercial or financial relationships that could be construed as a potential conflict of interest.
